# Mutation of a Threonine Residue in αD-β4 Loop of Cyt2Aa2 Protein Influences Binding on Fluid Lipid Membranes

**DOI:** 10.3390/toxins15020167

**Published:** 2023-02-19

**Authors:** Chontida Tangsongcharoen, Jose L. Toca-Herrera, Boonhiang Promdonkoy, Sudarat Tharad

**Affiliations:** 1 Faculty of Allied Health Sciences, Burapha University, Chonburi 20131, Thailand; 2 Institute of Biophysics, Department of Nanobiotechnology, University of Natural Resources and Life Sciences (BOKU), 1190 Vienna, Austria; 3 National Center for Genetic Engineering and Biotechnology, National Science and Technology Development Agency, Pathumthani 12120, Thailand; 4 Department of Biology, Faculty of Science, Burapha University, Chonburi 20131, Thailand

**Keywords:** Cyt2Aa2 protein, protein–lipid binding, membrane fluidity, AFM

## Abstract

Cyt proteins are insecticidal proteins originally from *Bacillus thuringiensis*. The lipid binding of the Cyt2Aa2 protein depends on the phase of the lipid bilayer. In this work, the importance of the conserved T144 residue in the αD-β4 loop for lipid binding on fluid lipid membranes was investigated via atomic force microscopy (AFM). Lipid membrane fluidity could be monitored for the following lipid mixture systems: POPC/DPPC, POPC/SM, and DOPC/SM. AFM results revealed that the T144A mutant was unable to bind to pure POPC bilayers. Similar topography between the wildtype and T144A mutant was seen for the POPC/Chol system. Small aggregates of T144A mutant were observed in the POPC and DOPC domains of the lipid mixture systems. In addition, the T144A mutant had no cytotoxic effect against human colon cancer cells. These results suggest that alanine replacement into threonine 144 hinders the binding of Cyt2Aa2 on liquid lipid membranes. These observations provide a possibility to modify the Cyt2Aa2 protein to specific cells via lipid phase selection.

## 1. Introduction

*Bacillus thuringiensis* (*Bt*) is a bacterium widely known for its insecticidal properties. This bacterium, which is a Gram-positive spore-forming aerobic bacterium, is naturally found in soil. During the sporulation phase of *Bt*, the active proteins Crystal (Cry) and Cytolytic (Cyt) proteins are produced as crystalline proteins [1]. In addition, during a vegetative phase, *Bt* insecticidal proteins are produced as vegetative insecticidal proteins (Vip) [2] and secreted insecticidal proteins (Sip) [3] as well. The *Bt* insecticidal proteins have been developed for use as biocontrol agents for a few decades. Particularly, Cry proteins are the most extensively used among *Bt* proteins as bioinsecticides for pest insect control in the form of either bioactive agents or transgenic plants [4,5]. Due to pest resistance against Cry proteins [6], other *Bt* proteins have been studied for their activity mechanisms in order to use them as a bioinsecticidal protein in parallel with Cry proteins. Cyt proteins are a good candidate because they do not need any receptor to interact with cell membranes [6,7,8]. In addition, Cyt proteins can synergize their activity with Cry proteins to overcome pest resistance [9,10,11]. 

Cyt proteins are classified into three classes, Cyt1, Cyt2, and Cyt3, based on their amino acid sequences [12]. In general, Cyt proteins are produced as inactive protoxins that require proteolytic activation to exert their activity [13]. After activation, Cyt proteins show cytolytic activity against insect cells, mammalian cells [7], and bacterial cells [14]. In vivo, Cyt proteins are mainly toxic to dipteran insects such as mosquitoes and black flies [15,16], and their toxicity can be extended to lepidopteran pests (*M. sexta* and *Plutella xylostella*) [17] and pea aphid (*Acyrthosiphon pisum*) [18] by protein engineering. The toxicity steps of Cyt proteins follow: (i) protein crystal solubilization, (ii) proteolytic activation, (iii) protein oligomerization/aggregation, and (iv) interruption of cell permeability [19,20]. The crucial step of cytolytic activity is protein oligomerization/aggregation [21,22]. In terms of conformational structure, the comparison between protoxin and active toxin is similar [23,24], so it has been proposed that the protein structures are altered with respect to lipid–lipid interaction. Cyt proteins are particularly preferable to unsaturated phospholipid [25]. In addition, cholesterol can promote protein–lipid membrane binding [26]. 

**Figure 1:**
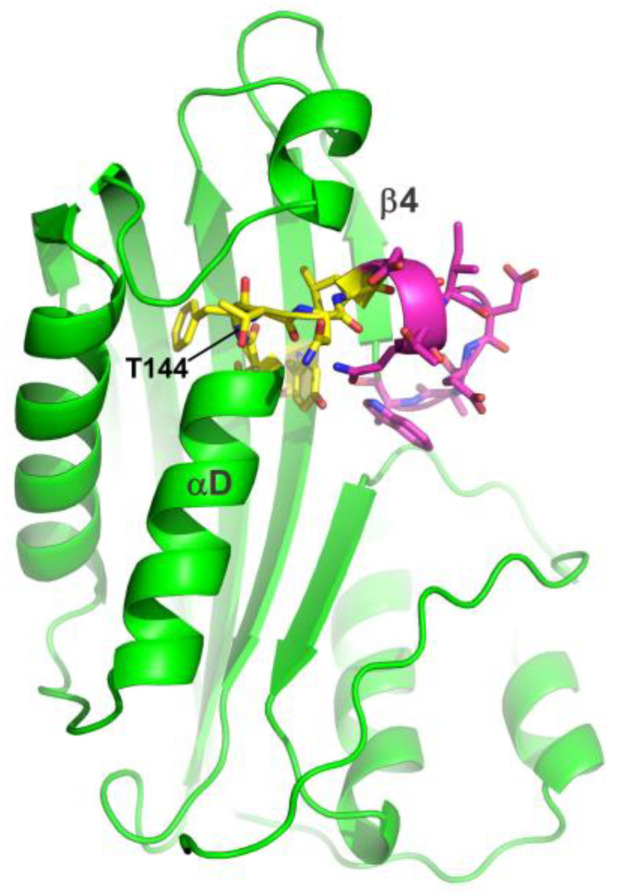
Three-dimensional crystal structure of Cyt2Aa2 protein.

In this study, we investigated the importance of the amino acid on protein–lipid interaction for the cytolytic Cyt2Aa2 protein originally produced from *B. thuringiensis* subsp. *darmstadiensis*. The full length of the Cyt2Aa2 protein is highly expressed in *Escherichia coli* [27], which facilitates the engineering of Cyt2Aa2 protein. The amino acid sequence alignment of the Cyt protein family shows highly conserved amino acids from T142 to L146 of the Cyt2Aa2 protein (Figure S1). These amino acid residues are located in the αD-β4 loop of the Cyt2Aa2 protein and have been proposed to play a role in protein–lipid binding (Figure 1). In particular, we investigated the importance of the replacement of the threonine 144 residue with alanine (T144A) in the protein–lipid binding process. This mutation (T144A) affects the ability of the Cyt2Aa2 protein to bind lipid membranes. Furthermore, the T144A mutation does not induce cytotoxicity against human colon cancer cells (proposed here because of their high membrane fluidity). This study links the lipid phase and the cell-specific relevance of Cyt2Aa2 protein activity. In the future, other mutations will be conducted in the αD-β4 loop to test the lipid binding ability of the Cyt2Aa2 protein.

The amino acid residues located in the αD-β4 loop of the Cyt2Aa2 protein (PDB: 1CBY) are shown in stick. The highly conserved amino acids are presented in yellow and the position of T144 is indicated. The magenta residues are the partially conserved amino acids. The image was generated using the PyMOL program.

## 2. Results and Discussion

### 2.1. Fluidity of Unsaturated POPC in the Hybrid Lipid Bilayers

The lipid components of the cell membrane, unsaturated phospholipid (POPC or DOPC), saturated phospholipid (DPPC), sphingomyelin (SM), and cholesterol (Chol) were mixed in order to form lipid bilayers with different phases as shown in Table 1. AFM experiments were conducted to investigate the lipid phase separation, lipid movement, and bilayer structure of POPC or DOPC. The lipid bilayer of POPC was successfully formed on the silica surface via lipid vesicle fusion, revealing a smooth surface with a roughness surface (a root mean square roughness, Rq) of 0.11 nm (Figure S2). Accordingly, the observation of POPC movement (liquid disordered phase, l_d_) was problematic.

**Figure 2:**
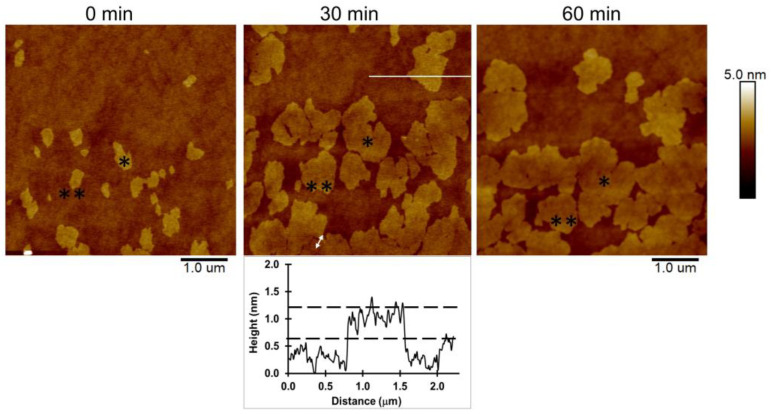
AFM sequence image showing DPPC domain reorganization over time of 1:1 DPPC/POPC bilayers.

The mixtures of saturated and unsaturated lipids were able to form phase separations [28,29]. The lipid bilayer of 1:1 DPPC/POPC exhibited lipid phase separation between the solid phase (S_o_) of the DPPC-enriched domain and the liquid disordered phase (l_d_) of the POPC-enriched domain, and its surface property is revealed differently in the AFM phase image (Figure S3). The height topographic image shows that the DPPC-enriched domains are thicker than the POPC-enriched domain by about 0.5–1.0 nm (Figure 2). Furthermore, the time sequence images of the 1:1 DPPC/POPC bilayer show the changes of DPPC domain shape with time. The size of the DPPC domains (asterisks) is significantly larger, from 0.241 μm^2^ to 1.54 μm^2^ and 1.90 μm^2^ after 30 and 60 min of lipid bilayer formation, respectively. An expanding domain was proposed due to the merging of DPPC lipid. Moreover, the DPPC domains moved close to each other. The double-ended arrow indicates the distance between the two DPPC domains of about 300 nm when these two domains contacted each other within 30 min. It seems that the DPPC domain (solid phase) is similar to an iceberg floating on a POPC ocean (liquid disordered phase). The changes in shape and size of the DPPC domains are related to the movement of the unsaturated POPC as a fluid lipid in the hybrid lipid bilayers. Hence, the fluidity of the lipid membrane can be observed during protein binding. 

Lipid bilayers were formed via the lipid vesicle fusion method. Lipid vesicle solutions were incubated on silicon wafers for 10 min. The lipid bilayer was rinsed with PBS buffer before AFM imaging. The topographic images were visualized using tapping mode AFM with a scanning rate of 1–2 Hz. DPPC domains are shown with asterisks and the distance between the domains is indicated by a double-ended arrow. The white large line without arrows in the 30 min image depicts the height profile (shown below). 

### 2.2. Binding of Cyt2Aa2 Mutant on Cholesterol-Embedded Lipid Bilayers

**Figure 3:**
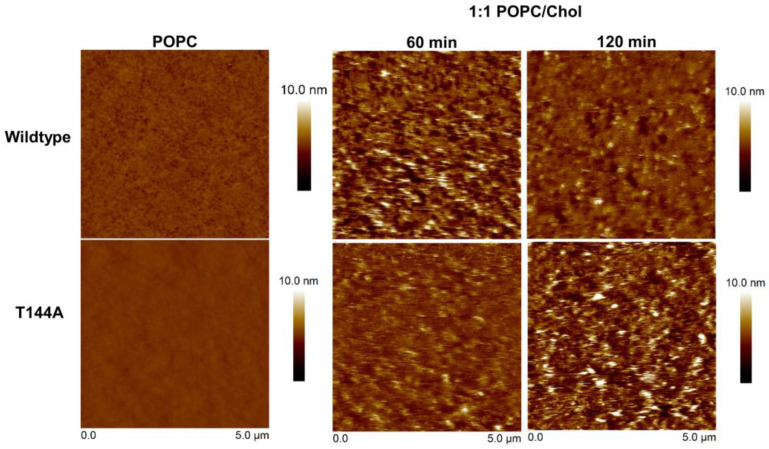
Protein–lipid binding of the Cyt2Aa2 wildtype and the T144A mutant onto POPC/Chol lipid bilayers.

The effect of lipid fluidity on protein–lipid binding was studied for the T144A mutant compared to the wildtype. The protein solutions were incubated with the lipid membranes for 2 h and the surface topography of the protein–lipid layers was imaged. Figure 3 shows that the T144A mutant did not bind on the POPC bilayer, a liquid disordered phase with high fluidity revealing a smooth surface (Rq = 0.12 nm). On the contrary, the wildtype shows a rough surface with Rq = 0.278 nm, indicating protein binding over the lipid surface. Subsequently, cholesterol (Chol) was embedded into the lipid bilayers (Rq = 0.12 nm) to form a lipid bilayer with a liquid ordered phase that would move less [30]. The 1:1 POPC/Chol lipid bilayer encouraged the lipid binding of the T144A mutant. However, the hybrid protein–lipid layer caused by the binding of both the wildtype and T144A mutant on the POPC/Chol bilayer was distinct from the wildtype-POPC membrane binding (Figure 3). After 60 min of incubation, the wildtype exhibited an irregular surface with large holes (Rq = 1.34 nm), and it seems that the hybrid layer reorganized into a more smooth surface with Rq = 0.69 nm (but the holes still remained) at 120 min, determined by decreasing Rq. For the T144A mutant, an irregular surface was initially observed with Rq = 0.70 nm at 60 min, and, subsequently, holes were detected with Rq = 1.59 nm at 120 min, which revealed a similar topography to the wildtype. Although surface reorganization could be seen for the mutant (Rq = 0.88 nm at incubation time 180 min), the surface was not as smooth as that of the wildtype (Rq = 0.69 nm) (Figure S4). These findings suggest that cholesterol promotes the binding of T144A onto the lipid membrane due to a reduced fluidity of the lipid membrane [31], which could favor T144A mutant docking and further formation of protein complexes on lipid membranes. Remarkably, high cholesterol content (1:1 POPC/Chol) contributed to the alteration of the Cyt2Aa2 binding behavior. The binding of Cyt2Aa2 to the POPC molecule (a target lipid) may have interfered with the interaction between POPC and cholesterol [32]. Therefore, the hybrid layer reorganized, reaching its final state. 

Lipid bilayers were formed on the silicon wafer via lipid vesicle fusion. The protein solution (25 µg/mL) was incubated with the lipid bilayers in order to evaluate the Cyt2Aa2-lipid binding as time sequence. The topographic images were visualized by tapping mode AFM with scanning rate 1–2 Hz.

### 2.3. Binding of Cyt2Aa2 Mutant on Two Phase State Bilayers

**Figure 4:**
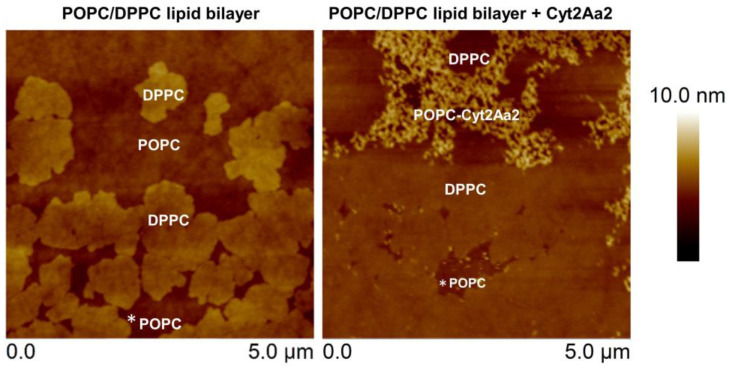
Protein–lipid binding of Cyt2Aa2 wildtype onto POPC/DPPC lipid bilayers.

**Figure 5:**
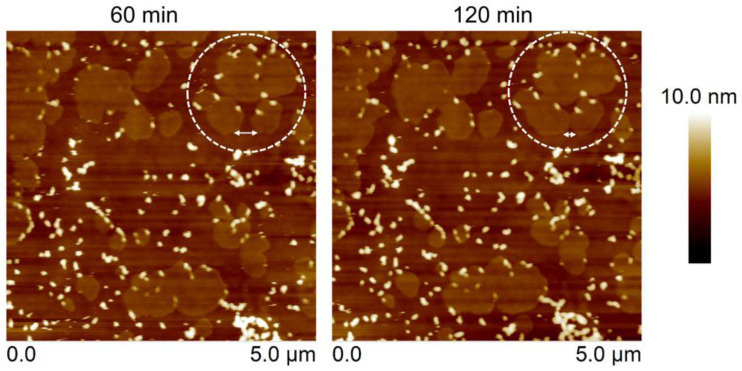
Protein–lipid binding of the T144A mutant onto POPC/DPPC lipid bilayers (time sequence).

Furthermore, protein–lipid binding was observed on POPC/DPPC bilayers, where the fluidity of POPC was indirectly influenced by the movement of DPPC domains (Figure 2). After protein overlaying, the wildtype could bind to the fluid POPC area in two-phase bilayers but not the more solid DPPC domains that exhibited an empty and smooth surface (Figure 4). After binding, the protein aggregates were thicker (lighter color) than the DPPC domains, suggesting that wildtype did not insert into the lipid membrane. Interestingly, the POPC domains around which DPPC clustered (asterisk) were bound by the protein less than the large POPC domain. Small aggregates were observed on these areas, giving clues that the properties of POPC were different between the two areas. The DPPC surrounding the POPC domains may act as a solid-phase domain, as the lipid molecules could move due to the blocking of the DPPC domain. In contrast, the T144A mutant could bind to the membrane, but it could not cover the surface as fully as the wildtype. The binding of T144A took place on the edge of the DPPC domains and the fluid POPC domain-forming protein aggregated (187 aggregates with average diameter of 105 nm). Though the incubation time was extended to 120 min, the binding of T144A mutant onto the lipid membrane did not significantly increase the number and size of T144A aggregates (196 aggregates with an average diameter of 114 nm) (Figure 5). During the protein binding process, the DPPC domains remained moving, and the distance between the DPPC domains was reduced from 350 nm to 150 nm (double-ended arrow) in 60 min. Remarkably, the T144A aggregates on DPPC edge domains moved together with the DPPC domains (in white circle). However, T144A aggregates on the POPC could not move. It is likely that protein binding inhibits the movement of POPC close to the binding area. 

After the lipid bilayers were formed, protein solution (25 µg/mL) was incubated with the lipid bilayers for 60 min to evaluate Cyt2Aa2-lipid binding. The topography of the protein–lipid layer was visualized via tapping mode AFM with a scanning rate of 1–2 Hz.

Lipid bilayers were formed on the silicon wafer via lipid vesicle fusion. T144A protein solution (25 µg/mL) was incubated with the lipid bilayers to evaluate Cyt2Aa2-lipid binding as a time sequence. The topography of the protein–lipid layers was visualized via tapping mode AFM with a scanning rate of 1–2 Hz. The double-ended arrows indicate the distance between the domains of 350 nm and 150 nm for 60 min and 120 min, respectively. 

**Figure 6:**
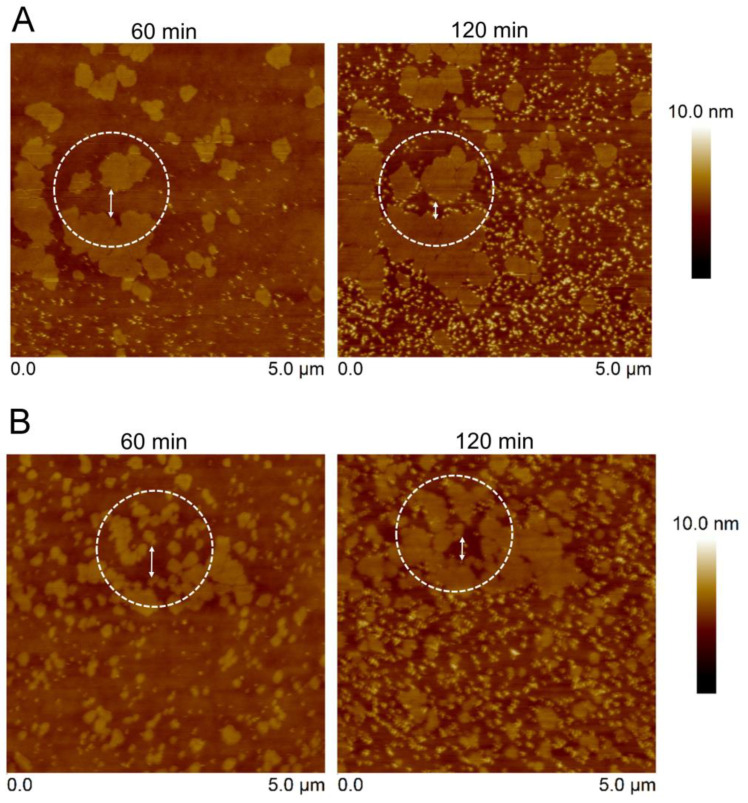
Protein–lipid binding of the T144A mutant onto POPC/SM and DOPC/SM lipid bilayers (time sequence).

For the sphingomyelin (SM)-phospholipid system, 1:1 POPC/SM and 1:1 DOPC/SM bilayers were prepared to form l_d_-S_o_ coexistence lipid phases. Both lipid mixtures revealed co-existing phases of l_d_ and S_o_ in the lipid bilayer in agreement with the lipid phase diagram [33,34]. In particular, the S_o_ domains of SM were distributed over the lipid surface, being higher than the POPC and DOPC enriched domains ca. 1.0 nm (Figure S5). After lipid bilayer exposure to the T144A mutant, protein aggregates were visualized on either the POPC-enriched domains (Figure 6A) or DOPC-enriched domains (Figure 6B) but not on the solid SM domains. T144A aggregates were found as individual complexes observed in both POPC/SM and DOPC/SM systems. The T144A binding increased the Rq from 0.42 nm to 0.59 nm for the DOPC/SM system, while Rq was changed from 0.44 nm to 0.75 nm for the POPC/SM system. Although the number of aggregates was increased, the T144A mutant could not fully cover the surface of these domains. In contrast, the wildtype occupied the whole areas of the POPC and DOPC domains, leaving the solid domains empty (Figure S6). Correspondingly, these lipid systems exhibited a result similar to the POPC/DPPC system. During protein incubation, the SM domains expanded and moved, reducing the distance between the domains with time. The SM domains could move between 180 nm and 170 nm in 60 min (double-ended arrow) for POPC/SM and DOPC/SM, respectively. These results suggest lateral movement of the lipid bilayers, implying lipid membrane fluidity. The presence of saturated DPPC and sphingomyelin (SM) in the POPC bilayer might provide different properties (i.e., the T144A mutant could not bind to the pure POPC bilayer). It is possible that the presence of SM and DPPC molecules among the POPC molecules in the lipid bilayer hindered the movement of POPC. In addition, the mixture of high melting lipids SM (T_m_ = 40 °C) and DPPC (T_m_ = 41 °C) reduced the fluidity of the lipid bilayer (less lateral diffusion) [34]. Hence, either SM or DPPC surrounding POPC had less fluidity (forming a nano-l_o_ domain), favoring the binding of T144A mutant and forming nano-protein complexes. The tendency towards T144A-lipid binding was expected to increase with the static SM and DPPC domains. This was thought to be feasible as SM and DPPC contain the same choline head group as POPC. However, T144A was unable to bind to the SM and DPPC domains. This suggests that other factors rather than lipid head group may play a role in the protein–lipid interaction (e.g., lipid membrane fluidity might influence Cyt2Aa2-lipid binding). In addition, the size of the lipid vesicle is affected by the binding of Cyt1A to the lipid membrane [35]. The T144A mutant preferably binds to a less fluid lipid membrane, such as the POPC/Chol system.

In terms of amino acids, the threonine 144 residue of Cyt2Aa2 plays an important role in lipid membrane binding. The hydroxyl group of the threonine side chain (-OH) possibly interacts with adjacent residues, especially N145 (Figure S7). In contrast, the alanine (containing a methyl group, -CH3) replacement for the T144 residue cannot provide lipid membrane binding, leading to insufficient binding to the fluid POPC bilayer. Moreover, the mutation of N145A also caused a loss of lipid membrane binding capability for reasons relating to cytolytic activity [36]. 

The lipid bilayers were formed on the silicon wafer via lipid vesicle fusion. T144A protein solution (25 µg/mL) was incubated with the lipid bilayers at certain times. The protein–lipid binding was observed via tapping mode AFM with a scanning rate of 1–2 Hz. The double-ended arrows indicate the distances between the SM domains in 1:1 POPC/SM (A) as 440 nm (60 min) and 260 nm (120 min). For DOPC/SM (B), the distances between the SM domains were 470 (60 min) nm and 300 nm (120 min). 

### 2.4. Cytolytic Activity of T144A Mutant on Cancer Cell Line

**Figure 7:**
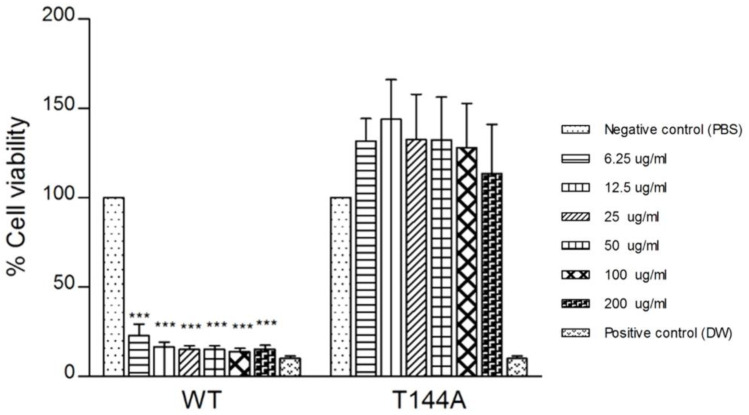
Cytotoxic effect of wildtype (WT) and T144A mutant proteins on human colon cancer cells (HCT116). *** *p* value < 0.001.

To extend our results on model systems, we explored the binding of T144A mutant with human colon cancer cells HCT116 (which have a fluid membrane). Thus, the cytotoxic effects of wildtype and T144A on HCT116 were investigated at different concentrations from 6.25 to 200 µg/mL. Cancer progression is associated with the modification of the membrane lipid composition leading to increasing membrane fluidity and protein dynamics [37,38]. The lipid composition of cancer cells has less cholesterol and a higher amount of unsaturated lipids than normal cells, which contain a high cholesterol content in their cell membranes [39]. A significant difference in cytotoxicity was observed when wildtype Cyt2Aa2 protein was tested against HCT116 cells. Wildtype protein exhibited strong cytotoxicity effects on HCT116 cells by about 85%, even at the lowest protein concentration (6.25 µg/mL). On the contrary, T144A mutant demonstrated no cytotoxicity against HCT116 cancer cells, even for the highest used protein concentration (200 µg/mL), see Figure 7. Compared to the cytotoxic effects of the Cyt2Aa2 protein, the cancer cells were observed to be more sensitive towards the wildtype than the T144A mutant. In agreement with this observation, previous studies on cancer cells revealed that some *Bti* proteins exert cytotoxic effects in human cancer cells [40]. However, in the present study, T144A was unable to damage or kill cancer cells. This may indicate that T144A does not bind to the fluid lipid membrane of cancer cells. The selective cytotoxicity of the wildtype and T144A mutant towards human colon cancer cells (HCT116) suggests that membrane fluidity can promote selective targeting between cancer and normal cells. Moreover, it was reported previously that the T144 residue is important for binding and complex formation in normal red blood cell membranes [36]. Therefore, it is worth noting that the wildtype and T144A hold promise for development as diagnostic markers to detect normal and cancerous cells.

Human colon cancer cells (HCT116) were treated with wildtype (WT) and T144A mutant proteins at different concentrations (6.25–200 µg/mL) for 24 h. The bar graphs show the percentage of cell viability assessed via MTT assay. Each experiment was performed independently in triplicate and results are presented as mean ± SEM. The *p* value was compared to the negative control (*** *p* value < 0.001).

## 3. Conclusions 

In conclusion, AFM results confirmed that the T144A mutant binds less effectively on the fluid lipid bilayer than the wildtype, which is in agreement with their cytotoxic effects on cancer cells. Hence, the alanine replacement of threonine 144 residue disables the Cyt2Aa2 protein binding to the liquid disordered bilayer. In particular, the T144 residue located in the αD-β4 loop seems to play an important role in the binding of the Cyt2Aa2 protein with lipid membranes. Furthermore, the modification of amino acid residues in the αD-β4 loop will be investigated for binding specific not only to the lipid phase but also the lipid charge.

## 4. Materials and Methods

### 4.1. Protein Preparation

The recombinant plasmid pGEM-Cyt2Aa2 containing the *Cyt2Aa2* gene was used as the template for the construction of the T144A mutant via PCR-based site-directed mutagenesis. Both recombinant pGEM-Cyt2Aa2 plasmids of wildtype and T144A were transformed in *E. coli* JM109. The changes in DNA sequence of the T144A recombinant plasmid were verified via DNA sequencing in the previous report [36]. Subsequently, Cyt2Aa2 protein from *Bacillus thuringiensis* subs. *darmstadiensis* was expressed in *Escherichia coli* as previously described by B. Promdonkoy [27]. In brief, both Cyt2Aa2 wildtype and T144A mutant proteins were expressed under lac promotor inducing with 1 mM IPTG (Isopropyl β-D-1-thiogalactopyranoside) (ThermoScienctific, Waltham, MA, USA) for 6 h at 37 °C. The cultured cells were collected and lysed via ultrasonication. Then, protein inclusions were harvested via centrifugation at 12,000× *g* for 10 min. The protein inclusions were resuspended and repeatedly sonicated in a washing buffer (50 mM Tris-base and 10 mM KCl, pH 7.5) plus 0.01% (*v*/*v*) Triton X-100. Subsequently, the protein inclusions were collected via centrifugation at 12,000× *g* for 10 min and washed twice with sterile distilled water. 

The partially purified inclusions of Cyt2Aa2 wildtype and T144 mutant were solubilized in 50 mM carbonate buffer, pH 10.0 at 30 °C for 1 h. The soluble Cyt2Aa2 proteins were collected via centrifugation at 10,000× *g* for 10 min. Then, Cyt2Aa2 proteins were activated by 2% (*w*/*w*) chymotrypsin at 30 °C for 2 h. After protein solubilization and activation, the purity of Cyt2Aa2 wildtype and T144 mutant proteins was determined via SDS-PAGE (Invitrogen, Waltham, MA, USA) (Figure S8) and protein concentration was determined via UV adsorption with extinction coefficient value of 1.16 (mg/mL)^−1^cm^−1^. 

### 4.2. Lipid Vesicle Preparation 

1-palmitoyl,2-oleoyl-sn-glycero-3-phosphocholine (POPC), 1,2-dioleoyl-sn-glycero-3-phosphocholine (DOPC), 1,2-dipalmitoyl-sn-glycero-3-phosphocholine (DPPC), chicken egg yolk sphingomyelin (SM), and cholesterol (Chol) were purchased from Sigma-Aldrich (Darmstadt, Germany). The lipids were mixed in chloroform with desired lipid ratios (Table 1). The lipid films were formed by slowly removing the solvent under a gentle nitrogen stream for 1 h. PBS solution, pH 7.4 (Sigma-Aldrich, Darmstadt, Germany) was added to the lipid films. In order to form lipid vesicles, the lipid films were incubated with PBS at above the melting transition temperature (T_m_) for 2 h and intermittently vortexed during incubation. Large unilamellar vesicles (LUVs) of POPC and POPC/Chol vesicles were produced by repeatedly pressing through a polycarbonate membrane 21 times at room temperature with a mini-extruder (Avanti, Birmingham, AL, USA). The sizes of the POPC and POPC/Chol vesicles were approximately 120–130 nm with a polydispersity index (PDI) of about 0.15. The LUVs of the POPC/DPPC, POPC/SM and DOPC/SM vesicles were obtained via tip sonication with a 50% duty cycle for 10 min (Branson sonifier, St. Louis, MO, USA). The sizes of the POPC/DPPC, POPC/SM and DOPC/SM vesicles were approximately 95–105 nm with a PDI of about 0.39. The vesicle sizes were determined via dynamic light scattering with a Zetasizer Nano ZS (Malvern Instrument, Malvern, Worcestershire, UK). The lipid vesicles were kept at temperatures higher than Tm and used within a week.

### 4.3. Anticancer Activity Assay

Human colon cancer cell line HCT116 (ATCC CCL-247) was purchased from American Type Culture Collection (ATCC, USA). Cells were grown in Dulbecco’s modified Eagle’s medium with a high glucose concentration (DMEM–HG; Gibco, Waltham, MA, USA) supplemented with 10% (*v*/*v*) fetal bovine serum (FBS; Gibco, Waltham, MA, USA), maintained at 37 °C with a 5% CO_2_ incubator. 

HCT116 cells (1 × 10^4^ cells/well) were cultured in 96-well culture plates for 24 h. The culture media was removed, and cells were then treated with 100 µL of various concentration of Cyt2Aa2 proteins in 3 replicates for 24 h before determining cell viability via MTT assay. Each Cyt2Aa2 wildtype and mutant T144A protein was prepared in medium to a final concentration of 200 µg/mL and diluted by two-fold from 200 µg/mL to 6.25 µg/mL. Meanwhile, 1x PBS (pH 7.4) was used as a negative control. After 24 h of incubation, 12.5 µL of 4 mg/mL MTT dye (Invitrogen, Waltham, MA, USA) was added to each well and incubated at 37 °C for 1 h in the dark to generate formazan crystals. Culture media were removed. To dissolve the crystal, 100 µL of DMSO was added and incubated further for 1 h. The absorbance was measured at 570 nm using a plate reader spectrophotometer (VersaMax, San Jose, CA, USA). This assay was performed in three independent experiments. The percentage of cell viability was determined by comparing the absorbance with that of the negative control (100% cell viability).

### 4.4. Atomic Force Microscope (AFM) Imaging

The AFM images were taken in tapping mode with a JV-scanner (NanoScope V controller) (Bruker, Billerica, MA, USA) mounting with a DNP-S10 AFM probe with a nominal spring constant of 0.24 N/m (Bruker, USA) at a scan rate of 1–2 Hz. Prior to lipid bilayer formation, the 1 cm × 1 cm silica wafers (IMEC, Leuven, Belgium) were cleaned in 2% (*w*/*w*) SDS solution with sonication for 15 min, rinsed with ultrapure water, and dried under a N_2_ stream. Finally, the surface was treated with plasma cleaner (Diener electronic, Ebhausen, Germany). The silica substrate was placed in the liquid chamber and sealed with an O-ring. To form the lipid bilayers, 0.1 mg/mL of lipid vesicle solution was flowed over the silica surface and incubated for at least 10 min. Afterwards, 25 µg/mL of Cyt2Aa2 protein was introduced into the chamber to expose the protein to the lipid bilayers. The AFM images were processed and analyzed with the Nanoscope program and the surface area was evaluated using the Image J program.

## Figures and Tables

**Table 1:** Lipid bilayers with different lipid compositions.

Molar Ratio of Lipid Compositions	Lipid Phase
POPC	Liquid disordered phase (l_d_)
1:1 POPC/Chol	Liquid ordered phase (l_o_)
1:1 SM/ POPC	Liquid disordered–solid phases (l_d_-S_o_)
1:1 SM/ DOPC	Liquid disordered–solid phases (l_d_-S_o_)
1:1 DPPC/POPC	Liquid disordered–solid phases (l_d_-S_o_)

## Data Availability

Not applicable.

## Supplementary Materials

The following supporting information can be downloaded at: https://www.mdpi.com/article/10.3390/toxins15020167/s1, Figure S1: Amino acid sequence alignment of Cyt2Aa2 with Cyt protein family and VVA2 protein; Figure S2: Lipid bilayer formation of POPC and 1:1 POPC/Chol; Figure S3: Lipid phase separation in 1:1 POPC/DPPC bilayer; Figure S4: AFM image of Cyt2Aa2 protein binding on the lipid bilayer of 1:1 POPC/Chol; Figure S5: Lipid bilayer formation of 1:1 POPC/SM and 1:1 DOPC/SM; Figure S6: Interaction of Cyt2Aa2 wildtype with 1:1 POPC/SM and 1:1 DOPC/SM bilayers; Figure S7: Location of threonine 144 residue in αD-β4 loop of Cyt2A protein. Figure S8. SDS-PAGE analysis of solubilized and activated Cyt2Aa2 wildtype and T144A mutant.
