# Supplementary material for: Mutation of a Threonine Residue in αD-β4 Loop of Cyt2Aa2 Protein Influences Binding on Fluid Lipid Membranes
Source: Toxins (Basel). 2023 Feb 19;15(2):167. doi: 10.3390/toxins15020167 (PMC9967203; doi:10.3390/toxins15020167)
Supplement: Supplementary file 1 [file toxins-15-00167-s001.zip › toxins-2131997-supplementary.pdf]

# Mutation of a threonine residue in αD-β4 loop of Cyt2Aa2 protein influences binding on fluid lipid membranes

**Chontida Tangsongcharoen[1], Jose L. Toca-Herrera[2], Boonhiang Promdonkoy[3] and Sudarat Tharad[4,*]**

[1] Faculty of Allied Health Sciences, Burapha University, Chonburi 20131, Thailand
[2] Institute of Biophysics, Department of Nanobiotechnology, University of Natural Resources and Life Sciences (BOKU), 1190 Vienna, Austria
[3] National Center for Genetic Engineering and Biotechnology, National Science and Technology Development Agency, Pathumthani 12120, Thailand
[4] Department of Biology, Faculty of Science, Burapha University, Chonburi 20131, Thailand

## Supporting data

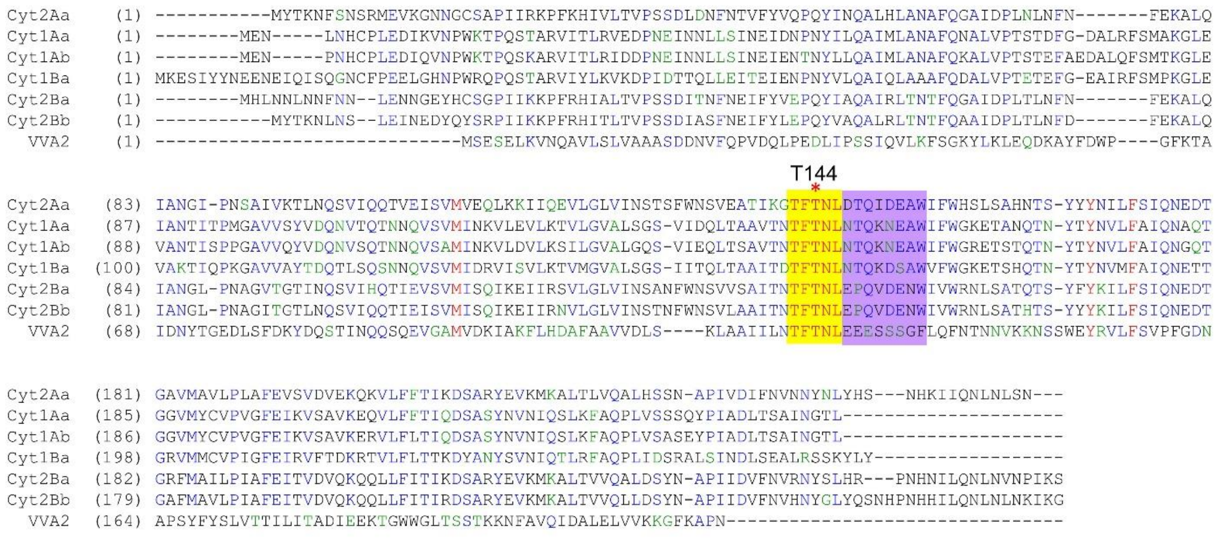


**Figure S1. Amino acid sequence alignment of Cyt2Aa2 with Cyt protein family and VVA2 protein.**

The T144A residue is conserved between Cyt protein family and VVA2 protein. The highly conserved amino acids are shaded in yellow while the partially conserved amino acids are shaded in magenta. The red asterisk indicates the T144 position.

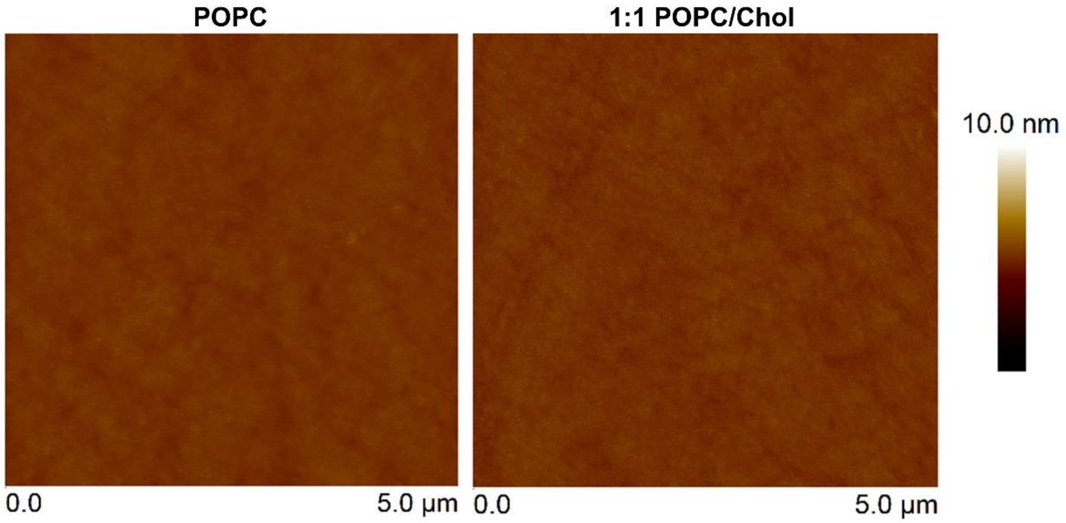


**Figure S2. Lipid bilayer formation of POPC and 1:1 POPC/Chol.**

Lipid bilayers were formed by lipid vesicle fusion method. Vesicle solutions (0.1 mg/ml) were incubated on silicon wafers for 10 minutes. The intact vesicles were flushed from the system before AFM imaging. AFM scanning was carried out in tapping mode with scanning rate 1-2 Hz.

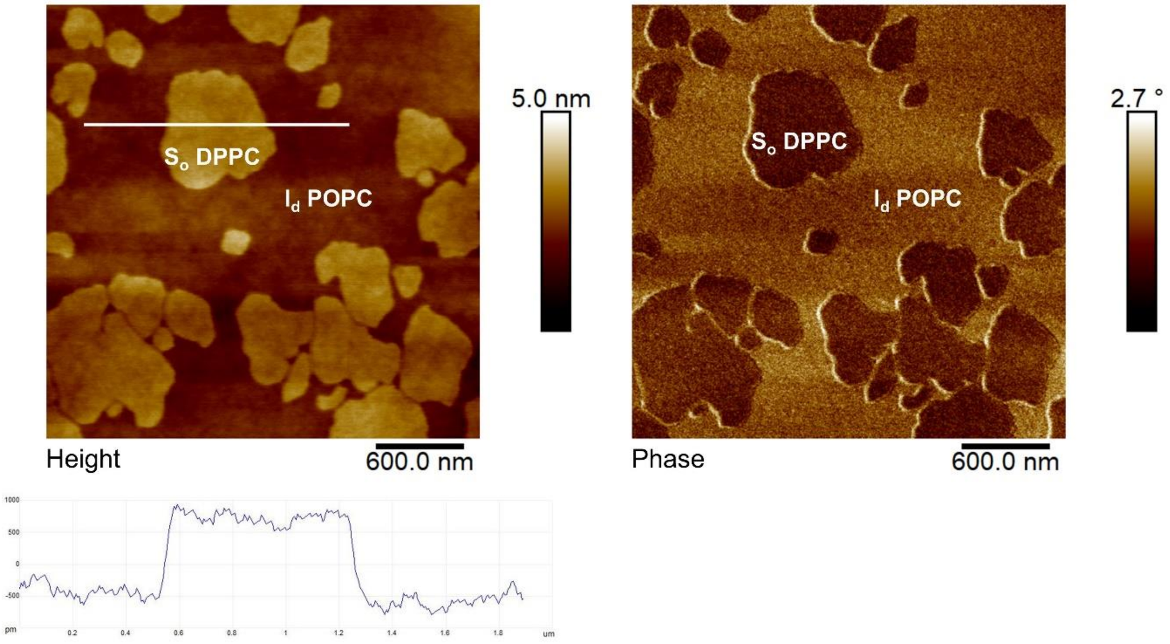


**Figure S3. Lipid phase separation in 1:1 POPC/DPPC bilayer.**

Lipid bilayer was formed by lipid vesicle fusion method. Vesicle solutions (0.1 mg/ml) were incubated on silicon wafers for 10 minutes. The intact vesicles were flushed from the system before AFM imaging. AFM scanning was carried out in tapping mode with BL-AC40TS cantilever (Olympus, Japan). The images were visualized with scanning rate of 1-2 Hz. Height image (left) and phase image (right). The white line on the height image depicts the height profile (shown below).

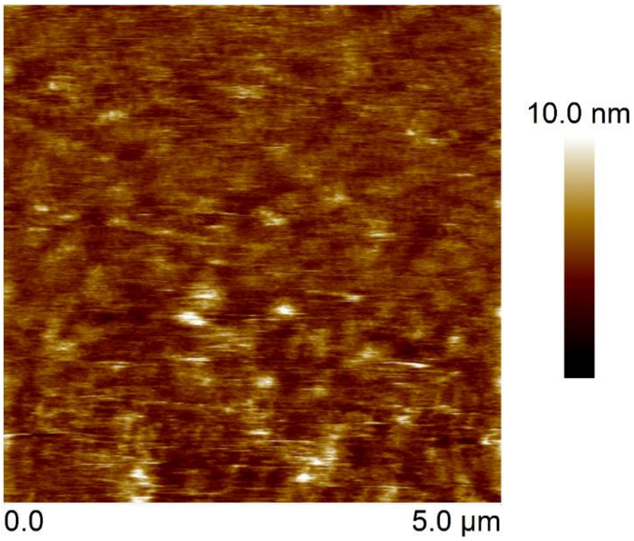


**Figure S4. AFM image of Cyt2Aa2 protein binding on the lipid bilayer of 1:1 POPC/Chol.**

Once the lipid bilayer was formed, Cyt2Aa2 protein (25 μg/ml) was introduced into the fluid cell chamber and incubated with the lipid membrane for 180 minutes. AFM images were collected in tapping mode with scan rate of 1–2 Hz.

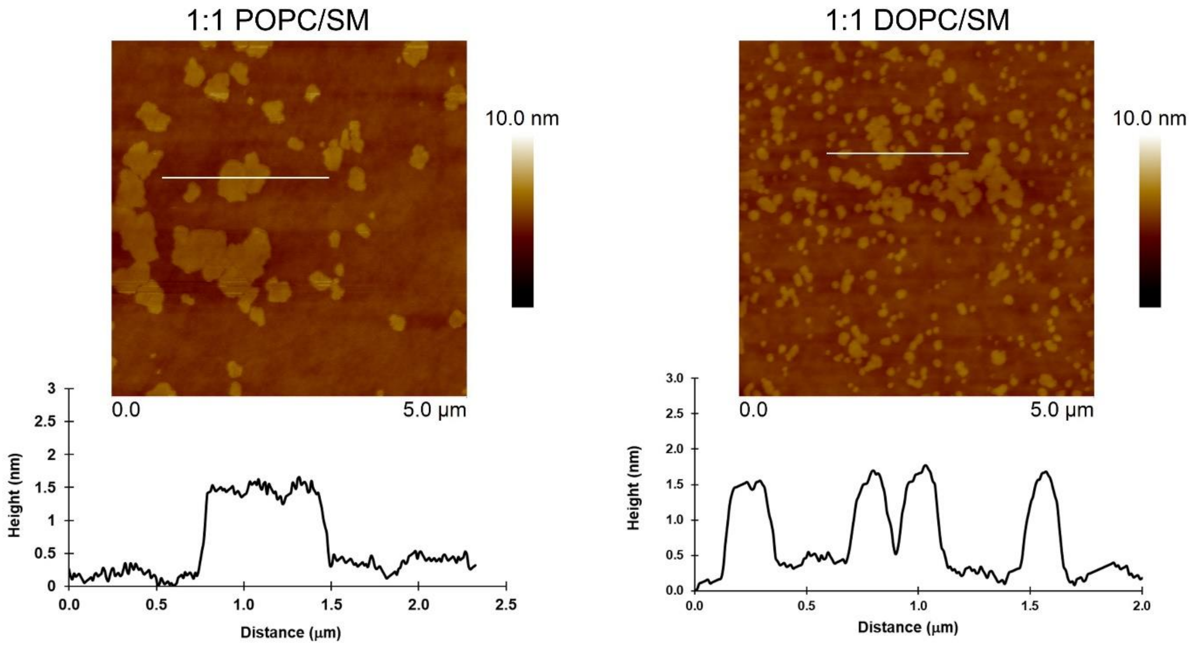


**Figure S5. Lipid bilayer formation of 1:1 POPC/SM and 1:1 DOPC/SM.**

Lipid bilayers were formed by lipid vesicle fusion method. Vesicle solutions (0.1 mg/ml) were incubated on silicon wafers for 10 minutes. The intact vesicles were flushed from the system before AFM imaging. AFM scanning was carried out in tapping mode with scanning rate 1-2 Hz. The white line corresponds to the cross section evaluation (height profile) of SM domain (see below the height profile versus the distance).

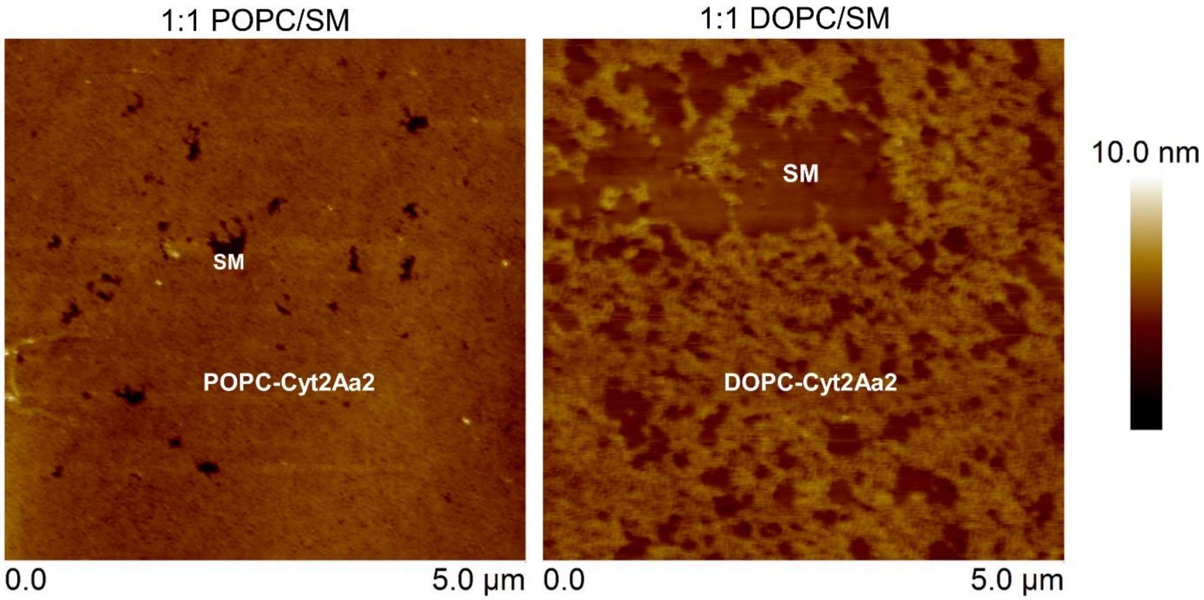


**Figure S6. Interaction of Cyt2Aa2 wildtype with 1:1 POPC/SM and 1:1 DOPC/SM bilayers.**

Once the lipid bilayers were formed, 25 µg/ml of Cyt2Aa2 wildtype was exposed to the bilayers for 1 hour. The images were obtained in AFM tapping mode with scanning rate of 1-2 Hz.

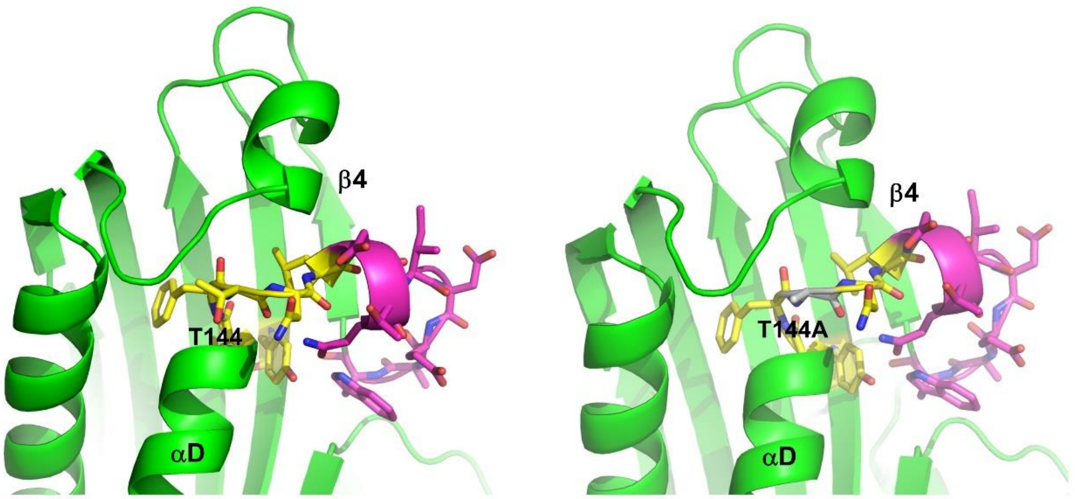


**Figure S7. Location of threonine 144 residue in $\alpha$D-β4 loop of Cyt2A protein.**

Three dimensional structure of Cyt2A protein (PDB 1CBY) is generated by PyMOL program. The high conserved amino acids of Cyt protein family are shaded with yellow whereas partially conserved amino acids are presented with magenta (left image). The replacement of the 144 residue with alanine is shown in a grey residue (right image).

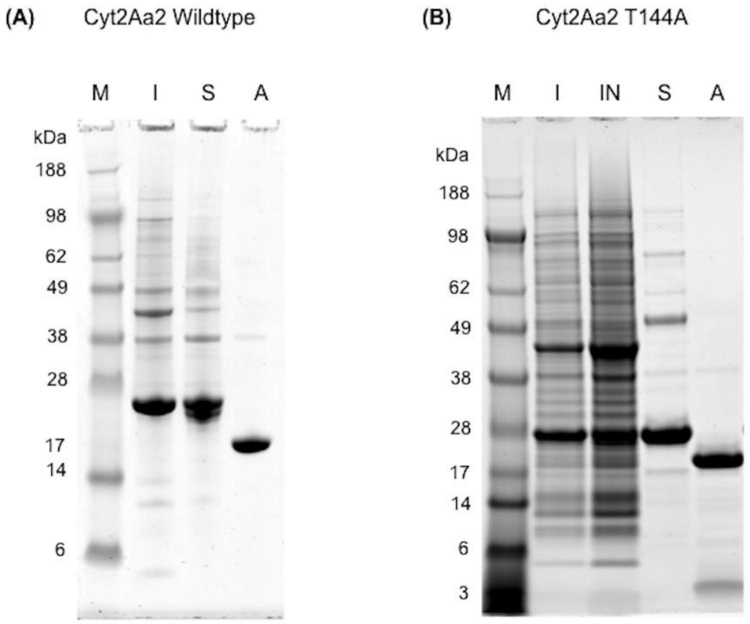


**Figure S8. SDS-PAGE analysis of solubilized and activated Cyt2Aa2 wildtype and T144A mutant.**

(A) Cyt2Aa2 wild type protein inclusion (I) was solubilized in 50 mM carbonate buffer, pH 10.0 for 1 hour at 30°C. Supernatant containing soluble protein (S) was separated by centrifugation. The soluble protoxin was activated (A) with chymotrypsin for 2 hours at 30°C.

(B) Cyt2Aa2 T144A protein inclusion (I) was solubilized in 50 mM carbonate buffer, pH 10.0 for 1 hour at 30°C. Insoluble protein (IN) and soluble protein (S) were separated by centrifugation. The soluble protoxin was activated (A) with chymotrypsin for 2 hours at 30°C.
